# LR11/SorLA and its role in cardiovascular disease

**DOI:** 10.1016/j.ijcha.2026.101982

**Published:** 2026-07-31

**Authors:** Meander Kloosterman, Ranitha Vongpromek, Edith C.H. Friesema, Wichor Bramer, Adrie J.M. Verhoeven, Monique T. Mulder

**Affiliations:** a Department of Internal Medicine, Laboratory of Vascular Medicine, Erasmus University Medical Center, Rotterdam, the Netherlands; b Medical Library, Erasmus University Medical Center, Rotterdam, the Netherlands

**Keywords:** SORL1 gene, sLR11, SORLA, Peripheral vasculature, Biomarker

## Abstract

Cardiovascular diseases account for the highest morbidity worldwide. LR11 (also called SorLA), an LDL receptor family member characterized as a sorting receptor, was initially identified in the brain and has a causative role in the development of Alzheimer's disease. However, LR11, and its circulating shed isoform, sLR11, are also associated to cardiovascular diseases and risk factors for atherosclerosis, such as obesity and diabetes. In the current narrative review, we discuss that elevation of plasma sLR11 levels can result from different forms of vascular injury, but also plays a role in subsequent vascular remodeling. We provide an overview of the mechanisms whereby LR11 promotes vascular remodeling and thereby atherosclerosis and how it could be involved in obesity and diabetes. Furthermore, we discuss the possibilities of (s)LR11 as a biomarker and therapeutic target for cardiovascular diseases.

## Highlights

• The mosaic receptor LR11 (aka SorLA) is known to be involved in Alzheimer's disease.
• LR11 in the circulation is increased in patients with some kind of vascular distress.
• LR11 induces vascular smooth muscle cell migration and monocyte recruitment in vitro.
• LR11 increases plasma triglyceride-rich lipoproteins and visceral adiposity.
• Vascular LR11 effects may lead to adverse vascular remodeling and atherosclerosis.

## Graphical abstract

**Figure:**
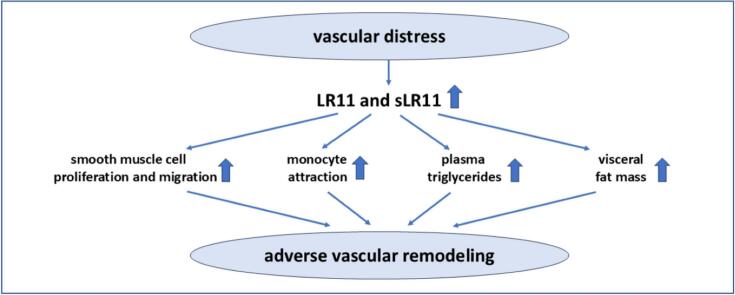

## 1 Introduction

Cardiovascular diseases (CVD) are the leading cause of death worldwide, and annually approximately 16 million people die as a result of ischemic heart disease or stroke [1], [2]. Ischemic heart disease and stroke are mainly caused by atherosclerotic plaque formation in the vascular wall of the coronary and carotid arteries, respectively. It is generally accepted that atherosclerosis development starts with inflammation of the endothelial layer. Subsequently, monocytes will infiltrate into the intima and vascular smooth muscle cells (SMCs) will proliferate and migrate from the media to the intima, both leading to cholesterol loading and intimal thickening. Key players in the development and progression of atherosclerosis are members of the low-density lipoprotein (LDL) receptor family, as these receptors are involved in cholesterol uptake. The most studied family member is the LDL receptor itself, which endocytoses ApoB100- and ApoE-containing lipoproteins to regulate intracellular and plasma cholesterol levels. Dysfunction of this receptor is associated with familial hypercholesterolemia (FH), and a highlyincreased risk of CVD [3], [4], [5]. Mice deficient for the *ldlr* gene develop atherosclerotic plaques when fed a Western-type diet, therefore these mice are used as a model for FH and atherosclerosis research [6].

The LDL receptor relative with 11 binding repeats (LR11, also called sortilin-related receptor with LDLR A-type repeats or SorLA) is another LDL receptor family member that is of interest in relation to CVD. LR11 is expressed in the vascular wall particularly on SMCs [7]. The expression of LR11 was found to be increased in atherosclerotic lesions [7] and the *Sorl1* gene encoding LR11 has been mapped to a pro-atherogenic locus in mice [8]. Initially LR11 was thought to be predominantly expressed in the brain, with the highest expression in neurons [9], [10]. Evidence was obtained indicating a role in neurodegenerative diseases e.g. in the development of Alzheimer's disease (AD). Genomic wide association studies confirmed a genetic association between the human *SORL1* gene and AD [11], [12], [13], [14]. Loss-of-function variants of the *SORL1* gene strongly increased the risk for early and late onset AD [15], [16]. Involvement of LR11 in the pathogenesis of AD is now well established and has extensively been discussed by Willnow et al. [17] and Salasova et al. [18].

Less is known concerning the role of LR11 in the development of CVD and many features of LR11 in vascular- and other related tissues require further elucidation. Therefore, this review aims to provide an overview of the mechanisms by which LR11 may influence the pathogenesis of atherosclerosis and other cardiovascular-related diseases. Furthermore, the potential of measuring soluble LR11 (sLR11) levels in plasma to predict the risk of CVD and the potential of therapeutic targeting of LR11 will be discussed.

## 2 The molecular characteristics of LR11

In 1996, Yamazaki et al. [10] described LR11 for the first time. In the same period, Jacobsen et al. [9] reported the existence of LR11, though they named it SORLA, as it appeared to be involved in intracellular protein sorting. In humans, the gene encoding LR11, *SORL1*, is located on chromosome 11q23.3–24 [19]. LR11 is a 250 kDa transmembrane protein, initially detected in neurons. Studies on other human organs revealed expression of LR11 in the spleen, prostate, ovaries, thyroid and testis [9]. Because apolipoprotein (Apo) E was identified as a ligand for LR11 [20] and levels of LR11 protein were found to be increased in atherosclerotic lesions in animal models [7], a role for LR11 in CVD was proposed.

**Fig. 1:**
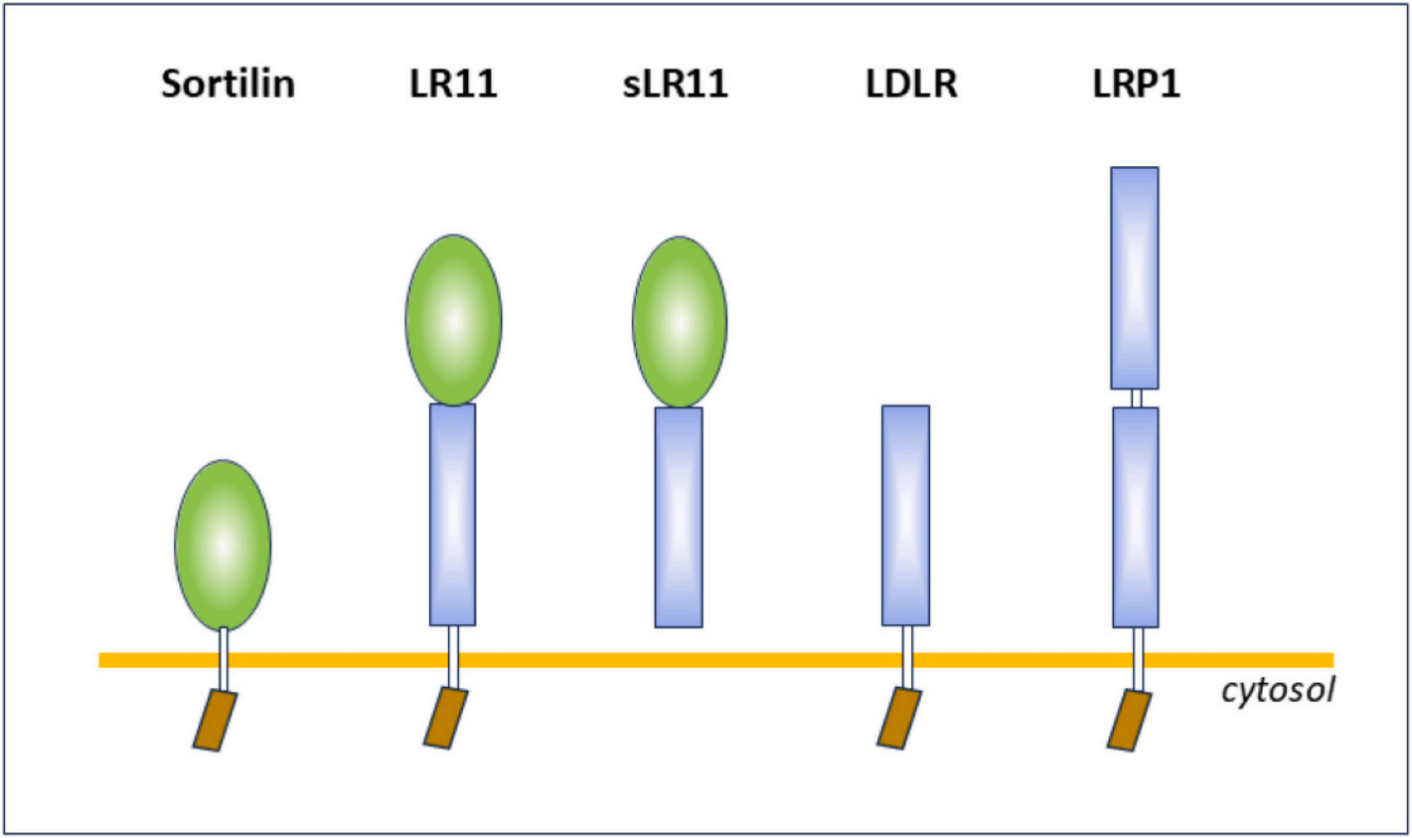
Schematic comparison of LR11 and sLR11 with related receptor proteins. LR11 is a mosaic receptor containing a VSP10p type ligand-binding domain similar to sortilin, as well as an LDL Receptor-type ligand binding domain. LRP1 contains multiple LDL Receptor-type ligand binding domains. These receptors have a cytoplasmic tail. In sLR11, the entire ectodomain of Lr11 is cut from the cytoplasmic tail by ADAM17, and becomes detached from the cell membrane.

LR11 is a mosaic receptor presenting similarities with multiple receptor families (Fig. 1). First, LR11 is related to the LDLR family, including the LDLR-related protein-1 (LRP-1), the very low-density lipoprotein receptor (VLDLR) and others. These receptors contain similar structural domains, though most LDLR ligands bind to the ‘LDLR ligand binding repeats’, which vary up to 40 residues. For instance, this domain has binding sites for receptor-associated protein (RAP) [9], ApoE [21], lipoprotein lipase (LPL) [22], ApoA-V [23] and amyloid precursor protein APP [24]. On the other hand, LR11 shows structural similarities with the vacuolar protein sorting 10 proteins (VPS10p), such as sortilin, which play a role in lipoprotein metabolism as well (reviewed in [25], [26]). Amyloid β peptide [27], tau [28] and interleukin-6 (IL-6) and IL-6 receptor [29] are among the proteins that bind to the VPS10p domain. VPS10p was identified in yeast where it is involved in the transport of newly synthesized peptidases from the *trans*-Golgi network (TGN) to the vacuole [30]. For LR11 a similar role has been hypothesized in intracellular sorting, which was supported by the observation that most of the protein is localized at the TGN instead of the plasma membrane [21], [31]. In newly synthesized LR11, the VPS10p domain is blocked with a pro-peptide, preventing ligands from binding. The pro-peptide is removed in the late TGN by a pro-peptide convertase, furin [32]. It is proposed that LR11 shuttles between the TGN, cell surface and endosomes/lysosomes to function as an intracellular sorting protein [33], [34]. It is now widely accepted that LR11 is an endosomal receptor that interacts with the multi-protein sorting complex retromer to traffic various proteins through the *endo*-lysosomal network [35]. Altered expression of LR11 and subsequently disturbed sorting of ligands (e.g. ApoE, APP, tau, IL-6) is thought to be the underlying cause of amyloidogenesis and neurodegeneration in AD.

Membrane-anchored LR11 is targeted by ADAM17 (also known as tumor-necrosis factor-alpha converting enzyme (TACE))-mediated ectodomain shedding resulting in the release of a soluble form of LR11 (sLR11) [36]. Besides the role of sLR11 in the progression of CVD, circulating sLR11 has been linked to parameters of CVD, such as intima-media thickness for atherosclerosis [37], [38], [39]. sLR11 could be an interesting target for further research of its function and as a biomarker in atherosclerosis progression and other CVD, which will be discussed below.

## 3 Levels of circulating sLR11 are associated with cardiovascular diseases

Since the group of Bujo developed an immunoassay to quantify the levels of sLR11 [40], many correlations with CVD have been found. To find papers for this narrative review with data on plasma sLR11 levels in patient populations, we performed a targeted literature search in EMBASE, Medline and COCHRANE databases on September 30th 2025. The search string was composed of (SORL1 or SORLA or LR11) and (cardiovascular or atherosclerosis) including synonyms and related words. We found 11 original papers in which plasma levels of sLR11 in patients were compared with a control group (Table 1), and 12 papers in which sLR11 levels were correlated with degree of disease (Table 2).

**Table 1:** Elevated sLR11 levels in various vascular diseases.

	Cases	n	Controls	n	sLR11 level (ng/mL)^a^	Additional findings	Ref
Wen et al. (2023)^b^	Women with pre-eclampsia (PE)	141	Normal pregnant women	60	Higher in PE (p < 0.001)	sLR11 higher in severe than mild PE (*p* < 0.001)	[53]
Watanabe et al. (2022)	Acute Kawasaki vasculitis	106	Healthy afebrile controls	18	14.9 [11.9–18.8] vs 11.9 [10.4–14.9] (*p* < 0.05)	sLR11 correlated with AST (r^2^ = 0.18; *p* < 0.0001) and bilirubin (r^2^ = 0.12; *p* = 0.003)	[42]
Joki et al. (2021)	Mitral regurgitation with pulmonary hypertension	10	Mitral regurgitation without pulmonary hypertension	24	14.4 ± 4.3 vs 9.9 ± 3.9 (*p* = 0.002)	sLR11 correlated with mean pulmonary artery pressure (r^2^ = 0.29, *p* < 0.001), transpulmonary pressure gradient (r^2^ = 0.18, *p* = 0.012) and pulmonary vascular resistance (r^2^ = 0.13, *p* < 0.05);	[48]
Ishida et al. (2019)^c^	Pre-eclampsia	14	Normal pregnancy; 3rd trimester	19	27.4 [20.0–32.0] vs 15.5 [13.0–19.3] (p < 0.001)	sLR11 in 3rd trimester are higher than in 1st and 2nd trimester; sRL11 correlates with TNFα in controls (r^2^ = 0.088; *p* = 0.036)	[52]
Harada et al. (2019)	Carotid artery stenosis	46	Healthy volunteers	52	12.2 ± 3.5 vs 9.0 ± 2.6 (*p* < 0.001)	sLR11 correlated with plaque score (r^2^ = 0.21; *p* < 0.01)	[41]
Jiang et al. (2019)	Carotid endarterectomy with intimal hyperplasia	10	Carotid endarterectomy without intimal hyperplasia	69	1.65 ± 0.25 vs 1.28 ± 0.28 (p < 0.001)	sLR11 predicts intimal hyperplasia after carotid endarterectomy	[45]
Unoki-Kubota et al. (2018)	Type 2 diabetes	70	Healthy controls	76	10.2 ± 4.2 vs 8.3 ± 2.1 (*p* = 0.002)	sLR11 associated with HOMA-IR (β = 0.26, *p* = 0.005), BMI (β = 0.24, *p* = 0.010) and age (β = 0.20, *p* = 0.027)	[43]
Watanabe et al. (2016)	Late Kawasaki vasculitis with coronary artery lesions	23	Late Kawasaki vasculitis without lesions	26	11.1 [9.3–13.9] vs 9.0 [7.7–10.1] (*p* = 0.01)	sLR11 correlated with hsCRP (r^2^ = 0.23, *p* < 0.01) and Lp(a) (r^2^ = 0.24, *p* < 0.01)	[55]
	Late Kawasaki vasculitis with coronary artery lesions	23	Cardiac patients without hemodynamic abnormalities	23	11.1 [9.3–13.9] vs 8.4 [7.1–10.2] (*p* = 0.001)		
Berk et al. (2016)	Overweight Type 2 diabetes	64	Nonobese healthy controls	64	15.4 [12.9–19.5] vs 10.2 [8.7–12.2] (p = 0.001)	Diet-induced weight loss of 10% reduced sRL11 to 13.3 [11.0–17.1]; p = 0.001)	[44]
Ogita et al. (2013)	Acute coronary syndrome (ACS)	50	Stable angina pectoris	50	9.88 ± 2.78 vs 8.18 ± 1.11 (*p* < 0.01)	In ACS patients, sLR11 correlated with hsCRP (r^2^ = 0.23; p < 0.01), WBC (r^2^ = 0.17; *p* < 0.05) and blood glucose on admission (r^2^ = 0.19; *p* < 0.01)	[56]
Takahashi et al. (2010)	Coronary artery disease with coronary stenosis	95	Coronary artery disease without coronary stenosis	55	4.9 ± 2.7 U vs 3.6 ± 1.8 U (*p* < 0.05)^d^	sLR11 associated with HbA1c (β = 0.21; p < 0.01)	[46]

Abbreviations: AST: serum aspartate aminotransferase activity; BMI: body mass index; HOMA-IR: homeostatic model assessment for insulin resistance; hs-CRP: high-sensitivity C-reactive protein; WBC: white blood cell count.

a : mean ± SD or median [IQR].

b : data extracted from abstract.

c : data estimated from article figure.

d : 1 U corresponds to 50 ng/ml [52].

**Table 2:** Associations with sLR11 levels.

	Study population	n	Dependent variable	Independent variable	Major finding	Results	Ref
Wen et al. (2023)^a^	Pre-eclampsia	141	Serum level of ESM-1 and AGE	sRL11 level	sLR11 level positively correlated with levels of ESM-1 and AGE	Model of sLR11 (≥11.65 ng/mL), ESM-1 (>2.14 ng/mL) and AGE (>57.38 ng/mL) predict adverse pregnancy outcome (AUC-ROC 0.947, sensitivity 97.3%; specificity 82.0%)	[53]
Watanabe et al. (2022)	Acute Kawasaki vasculitis	106	Response to IVIG therapy	sRL11 level responders (*n* = 85) 19.6 [13.0–24.9]; non-responders (*n* = 21) 14.3 [11.7–16.5] (*p* < 0.01)	sLR11 predicts non-response to IVIG treatment	AUC-ROC 0.77; cut-off 17.5 ng/mL, sensitivity 66.7% and specificity 78.8%	[42]
Joki et al. (2021)	Mitral valve replacement in left heart disease	34	Hospitalization for HF or cardiovascular death during follow-up of 5y	Low and high sLR11 group (*n* = 24 and 10 resp.) below and above 9.4	More events in the high sLR11 group (35% vs 0%)	HR was 1.06 (95%CI 0.90–1.26); log-rank p < 0.05	[48]
Suwa et al. (2020)	CAD patients after PCI	223	Composite CVD endpoints during follow-up of median 2884d	Low and high sLR11 group below and above median 10.84	More CVD events in the high LR11 group (log-rank, *p* = 0.003)	sLR11 associated with CVD events (adjusted HR 2.47; 95%CI: 1.29–4.92; *p* = 0.006)	[49]
Harada et al. (2019)	Carotid artery stenosis	27	Progression of stenosis during follow-up of 1y	Initial sLR11 level	sLR11 varies with stenotic fraction changes ranging from −6% to +6%	initial sRL11 correlated with stenosis progression (r^2^ = 0.22; *p* = 0.04)	[41]
Jiang et al. (2019)	Carotid endarterectomy	79	Intimal hyperplasia during at least 3mo follow-up	Initial sLR11 level	sLR11 predicts intimal hyperplasia after carotid endarterectomy	AUC-ROC 0.85; cut-off 1.44; sensitivity 90% and specificity 73.5%	[45]
Vongpromek et al. (2017)	Male asymptomatic FH patients	78	Aortic root calcification	sLR11 level	sLR11 is associated with presence of aortic root calcification (OR 1.31; 95%CI 1.03–1.66; *p* = 0.03)	sLR11 is associated with severity (OR 2.01; 95%CI 1.28–3.16; *p* = 0.002) of aortic root calcification	[51]
Jin et al. (2016)	Type 2 diabetes	165	Carotid Intima-media thickness	sLR11 level	sLR11 correlated with IMT (β = 0.008; 95%CI 0.000–0.017; *p* = 0.044)	Association is independent of fasting glucose and LDL-C	[38]
Ogita et al. (2016)	CAD patients after coronary intervention	436	Composite CVD endpoints during follow-up of median 2876d	Quartiles of sLR11 level	HR increased dose-dependently with sLR11 quartile (p for trend = 0.0077).	Adjusted HR for Q4 versus Q1 was 2.68; 95%CI 1.32–5.46; *p* = 0.008)	[50]
Vongpromek et al. (2015)	Statin-treated asymptomatic male FH patients	32	Carotid Intima-media thickness	sLR11 level	sLR11 correlated with IMT (β = 0.05; *p* = 0.001)	Association is independent of systolic blood pressure and LDL-C	[39]
Shimizu et al. (2014)	Patients undergoing G-CSF stimulation for BM stem cell collection	14	Circulating myeloid cells	sLR11 level	sLR11 enhances myeloid cell migration from BM and attachment to TNFα-treated endothelial cells	sLR11 correlates with WBC count at d1 of treatment	[60]
Ogita et al. (2014)	Stable angina pectoris treated with a drug-eluting stent	102	Restenosis after follow up of 240d	sLR11 level	sLR11 at 240d after stenting was an independent variable of restenosis (β = 0.21; *p* = 0.039), but baseline sLR11 was not	sLR11 initially increased at d14 and d60 but had returned to baseline at d240	[47]

Abbreviations: AGE: advanced glycation end products; AUC-ROC: area-under-the-curve of receiver-operating characteristic; BM: bone marrow; CAD: coronary artery disease; CVD: cardiovascular disease; d: day(s); ESM-1: endothelial cell-specific molecule-1; HR: hazard ratio; IMT: intima-media thickness; IVIG: intravenous immunoglobulins; OR: odds ratio; PCI: percutaneous coronary intervention; WBC: white blood cell count.

a : data extracted from abstract.

Reported plasma sLR11 levels generally range between 5 and 35 ng/mL (Table 1), with mean or median levels ranging from 8.3 till 11.9 ng/mL in populations of healthy controls [41], [42], [43], [44]. One study reported much lower plasma levels [45], whereas one older study reported sLR11 levels in units which when converted to ng/mL would give much higher levels than most other studies [46]. Whether these values reflect real differences among patient populations, or are due to differences in assay conditions, is unclear. Plasma sLR11 levels were shown to be elevated in a variety of vasculature-related diseases (Table 1), such as carotid and coronary artery stenosis, pre-eclampsia, Kawasaki vasculitis, type 2 diabetes and pulmonary hypertension. The pathophysiological mechanism of these diseases differ markedly, suggesting that elevation of circulating sLR11 levels results from the vascular stress imposed by arteriosclerosis, inflammation, metabolism or hypertension.

Plasma sLR11 levels were associated with the degree or severity of vasculature-related diseases (Table 2). sLR11 levels correlated with intima-media thickness in patients with dyslipidemia [37], patients with type 2 diabetes [38] and asymptotic male patients with familial hypercholesterolemia (FH) [39]. In patients with carotid stenosis, sLR11 levels correlated with plaque score [41]. Ogita et al. [47] found that sLR11-levels correlated with restenosis after coronary stenting. sLR11 levels were associated with long-term adverse cardiac events [48], [49], [50] such as cardiovascular death, non-fatal acute coronary syndrome and non-fatal stroke. This correlation between sLR11 levels and CVD endpoints appears to be dose-dependent, indicating that sLR11 levels could serve as a valuable biomarker for future disease-related cardiovascular events [41]. However, these studies are relatively small and largely observational, and larger prospective studies are needed to validate sLR11 as a potential biomarker.

Vongpromek et al. [51] revealed a correlation with aortic root calcification in statin-treated asymptotic male FH patients. The latter correlation indicates a role of LR11 in vascular calcification. Plasma sLR11 levels were also elevated in women suffering from pre-eclampsia [52], [53], and more in patients with severe than mild pre-eclampsia [53]. Plasma sLR11 levels were found to be associated with pulmonary artery hypertension and with mean pulmonary arterial pressure and pulmonary vascular resistance [54]. For some diseases, sLR11 levels were found to correlate with inflammation markers such as hsCRP [55], [56], TNFα [52] and AGEs [53].

sLR11 levels are higher in patients with type 2 diabetes (T2D), a risk factor for CVD, compared to healthy people [46]. Multiple studies have shown a positive correlation between sLR11 and HbA1c, [44], [46], [57], [58] which is an indicator of long term hyperglycemia. Berk et al. [44] showed that diet-induced weight loss in overweight and obese patients with T2D resulted in lower sLR11 levels and this was positively associated with lower HbA1c levels. Takahashi et al. [46] found a correlation between insulin resistance (HOMA-IR) and sLR11 levels. Moreover, they showed a positive correlation between sLR11 and plasma triglyceride rich lipoproteins (TGRLs) [46], which are a risk factor for CVD and which are generally increased in diabetes [59]. These results indicate that LR11 or sLR11 might affect the progression of diabetes via a mechanism that involves TGRLs, which will be discussed in section 5.3 below. However, these speculations require further investigation to elucidate a role of LR11 in the pathogenesis of diabetes.

## 4 Regulation of LR11 expression in the cardiovascular system

In the neural system, expression of LR11 has been shown to be increased by brain-derived neurotrophic factor BDNF [61] and by omega-3 fatty acids [62], but whether this also occurs in the cardiovascular system is unknown yet.

In SMCs isolated from rabbit aorta, the expression of LR11 is increased by treatment with platelet-derived growth factor-BB (PDGF-BB) [63]. PDGF-BB induces the migration of vascular SMCs [64], [65].

In SMCs isolated from mouse pulmonary arteries, the expression of LR11 is increased by culturing under hypoxic conditions, in parallel with an increase of hypoxia-induced factor (HIF)1α; knockdown of HIF1α abolished the effect of hypoxia on LR11 expression [53]. Hypoxia prevents the rapid degradation of HIF1α protein seen under normoxic conditions [66]. Hypoxia and chemically induced HIF1α activity also increased expression of LR11 and sLR11 in human monocytic U937 cells [67]. In the proximal promoter region of the human *SORL1* gene, a consensus binding site for HIF1α has been identified, to which HIF1α binds in U937 cells under hypoxia [67]. HIF1α is widely expressed also in the cardiovascular system [66], and hypoxia and HIF1α have been implicated in the etiology of several CVDs including atherosclerosis [66], [68], [69]. It is likely, therefore, that LR11 expression is also regulated by HIF1α in the cardiovascular system.

In the neuroblastoma cell line SH-SY5Y, the transcription factor Forkhead Box P1 (Foxp1) was found to bind to the minimal promoter of the *SORL1* gene [70]. Silencing of Foxp1 reduced levels of LR11 mRNA and LR11 protein in these cells [70]. Foxp1 is widely expressed in the vasculature, including vascular SMCs and endothelial cells (ECs) [71]. Foxp1 is a transcriptional mediator of proliferation and differentiation in different atherosclerosis relevant cell types, especially ECs and SMCs, and has been shown to exert anti-atherosclerotic effects in controlling coronary thrombus formation, atherogenesis and acute coronary syndrome [71]. Foxp1 has been shown to regulate the degradation rate of HIF1α and hence, HIF1α activity in murine cardiomyocytes [72]. It is possible, therefore, that Foxp1 regulates expression of the *SORL1* gene also in the cardiovascular system.

## 5 LR11 and atherosclerosis

As atherosclerosis is the most common cause of CVDs, such as ischemic heart disease and stroke, the mechanism with which LR11 may affect atherosclerosis will be further discussed. There are two major hypotheses concerning the role for LR11 in the pathogenesis of atherosclerosis: i) a direct effect of LR11 on the vessel wall and ii) an indirect effect via a modulatory role in the regulation of lipoprotein metabolism leading to increased levels of TGRLs, a risk factor for CVD. LR11 appears to be also involved in obesity which will be discussed as well.

### 5.1 Role for LR11 in vascular cell functioning: Smooth muscle cells

LR11 may modify the development of atherosclerosis directly by affecting vascular cell function. As mentioned, LR11 was shown to be upregulated in atherosclerotic lesions in the aorta of rabbits fed a high cholesterol diet [7], in the carotid arteries of rats subjected to balloon injury [7] and in the aorta of apoE knock out mice [72]. Kanaki et al. [7] revealed its presence mainly in the intimal SMCs and in medial SMCs at the medial-intimal border. In vitro work revealed that LR11 and sLR11 levels were elevated in proliferating SMCs, accompanied by increased secretion of sLR11 [7], [72]. Zhu et al. [73], [74] prepared SMC primary cultures from aortas of atherosclerotic rabbits and showed that migration and invasion activity of SMCs was linked to the expression level of LR11. Furthermore, sLR11 in conditioned medium of LR11-expressing cells appeared to induce migration and invasion of SMCs in vitro [73], [74]. Involvement of LR11 in SMC migration and proliferation in intimal thickening was also demonstrated in a mouse model of pulmonary arterial hypertension [49] and of cuff-induced vascular changes [37], [73]. In vitro experiments with the SMCs derived from LR11-deficient mice revealed reduced proliferation, compared to SMCs from wild-type mice [49]. Concluding, multiple studies support the hypothesis that LR11 is involved in vascular remodeling via its key role in vascular SMC migration and proliferation.

**Fig. 2:**
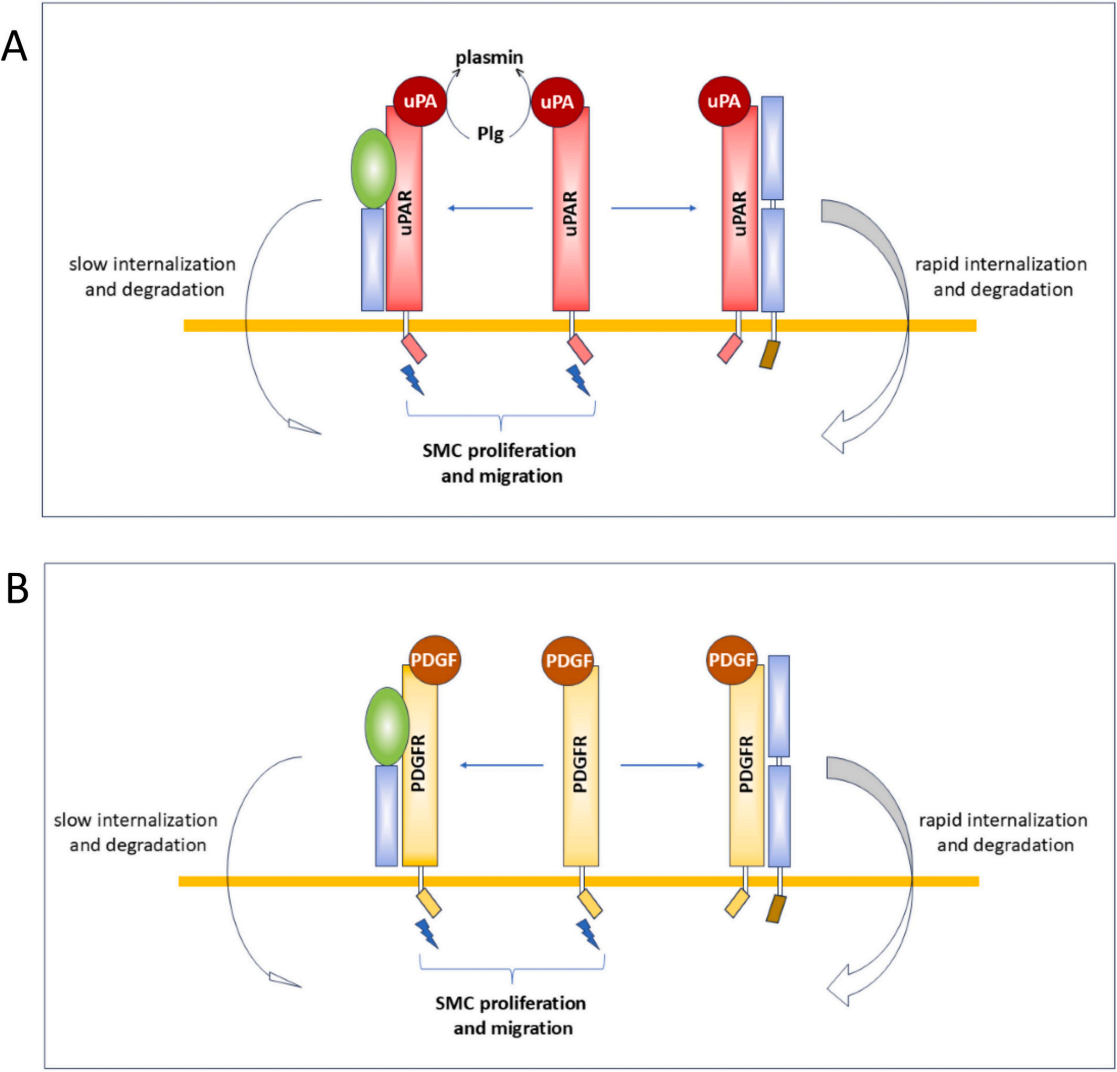
Competition between binding to LR11 and LRP1 leads to prolonged activity of uPA via uPAR (A) and PDGF-BB via PDGFR (B). Binding of uPAR and PDGFR to LR11 instead of to LRP1 slows internalization and intracellular degradation of these receptors. Why internalization of the complex with LR11 is slower than of the complex with LRP1 is unclear. Possibly, the LR11 ectodomain has become separated from the cytoplasmic tail which directs internalization [25] (Plg: plasminogen).

The mechanisms underlying the LR11-mediated SMC migration may involve the urokinase-type plasminogen activator receptor (uPAR), an established regulator of cell migration. In SMCs, elevated levels of LR11 were associated with an increased expression of the membrane-anchored receptor uPAR [74]. uPAR is known to be involved in the cleavage of plasminogen into plasmin via activation by uPA. Subsequently, the release of plasmin results in extracellular matrix degradation, which promotes SMC migration and invasion [75]. Alternatively, intracellular signaling of uPAR through Rac1 activation has been shown to be important for uPAR-mediated cell migration [37], [76]. Membrane-bound LR11, as well as sLR11, forms a complex with uPAR, and co-localize on the cell surface [73]. Gliemann et al. [77] showed that similar to LRP-1, LR11 binds to the ternary complex of uPAR, uPA and the type-1 inhibitor PAI—I. Binding of this complex to LRP-1 results in fast internalization and degradation of the uPA–PAI-I complex, whereas internalization and degradation of the complex is much slower when bound to LR11 [77]. Furthermore, LRP-mediated internalization and degradation of uPAR is inhibited by the interaction between LR11 and uPAR [73]. In the end, a higher expression of LR11 results in reduced internalization of uPAR and enhanced levels of the membrane-anchored receptor (Fig. 2A). This leads to an increased vascular SMC mobility through extracellular matrix degeneration and Rac1 mediated cytoskeleton reorganization [37], [73].

Alternatively, LR11 might enhance vascular SMC migration via platelet-derived growth factor-BB (PDGF-BB). PDGF-BB is known to be a potent chemoattractant for vascular SMCs and induces migration via the PDGF receptor (PDGFR) [64], [65]. Gliemann et al. [77] demonstrated that LR11 binds PDGF-BB, similar to LRP-1. Binding of LRP-1 to PDGF-BB generates co-localization of LRP with the PDGFR [78]. Binding of PDGF-BB to LR11 may result in elevated cell surface levels of PDGFR and reduced clearance of PDGF-BB (Fig. 2B) [77]. Subsequently, in combination with increased levels of uPAR, this could promote vascular SMC migration.

Another chemoattractant for SMCs, angiotensin II [79], might also be involved in vascular remodeling through LR11-mediated enhancement of SMC migration. In vitro assays with aortic SMCs from both LR11-deficient and wild-type mice demonstrated that Ang-II induced migration and attachment of the LR11 knockout cells to a collagen-coated surface was almost completely abolished. Membrane ruffling, which is associated with cytoskeleton reorganization and cell attachment, was enhanced in wild-type SMCs following pre-incubation with angiotensin II, but was abolished in LR11 knock-out SMCs or by blocking uPAR in wild-type SMCs. These results suggest that the presence of angiotensin II and elevated levels of LR11 and uPAR on intimal SMCs result in an increase in membrane ruffling, which enhances SMC attachment and eventually SMC migration [37].

### 5.2 Role for LR11 in vascular cell functioning: other vascular cell types

LR11 appears to be predominantly expressed on SMCs, yet LR11 may also affect other cell types involved in vascular remodeling, including endothelial cells and macrophages. Coronary artery endothelial cells (CAEC) also express LR11, but only under pro-atherogenic conditions, such as low shear stress and/or the presence of modified LDL [79]. Conditioned medium from human CAEC cultured under low shear stress conditions, which contained sLR11, induced vascular SMC migration in vitro [80]. These results indicate that in areas under low shear stress, vascular SMC migration may be further enhanced by the endothelial production of sLR11.

Induction of atherosclerosis by high-fat feeding and cuff treatment led to fewer lipid-loaded macrophages in the intima of LR11-knock out mice than in wild-type mice [81]. This suggests a role in foam cell formation, either of LR11 expressed by the macrophages [82], or of sLR11 released by the SMCs. Incubation of cultured macrophages with sLR11 increased uPAR levels on the cell surface and increased lipid accumulation via upregulation of scavenger receptors [81]. uPAR appears to play a role in monocyte adhesion and infiltration into the intima where differentiation to foam-cell macrophages occurs [81], [83], [84]. Therefore, sLR11 may be involved in monocyte recruitment, infiltration and foam cell formation in the vessel wall.

### 5.3 Interaction between LR11 and lipoprotein metabolism

LR11 may increase atherosclerotic risk by affecting PCSK9 activity [85]. Circulating PSCK9 binds to the LDL-receptor, which results in internalization, intracellular degradation and reduced cell-surface expression of the LDL-receptor. LR11 has been shown to bind circulating PSCK9 with high affinity, thereby inhibiting its inactivation [85]. An increased PCSK9 activity leads to reduced clearance of circulating LDL and thereby to elevated plasma LDL-cholesterol levels.

LR11 and sLR11 may also affect the development and progression of CVD indirectly via a modulatory role in the metabolism of triglyceride-rich lipoproteins (TGRLs). TGRLs include (β-)VLDL, chylomicrons, and their remnants, all carrying ApoE and ApoA-V in addition to ApoB100 or ApoB48 [86]. Increased plasma levels of TGRLs are an independent risk factor for CVD, even when patients are treated with statins that lower plasma LDL-cholesterol levels [87], [88], [89]. TGRLs are atherogenic due to enhanced arterial wall retention and subsequent inflammatory response and endothelial dysfunction [89]. TGRLs are associated with increased carotid intimal-medial thickness (cIMT), an indicator of atherosclerosis [90], [91]. Thus, strict management of plasma TGRL levels is important to maintain a healthy cardiovascular system.

**Fig. 3:**
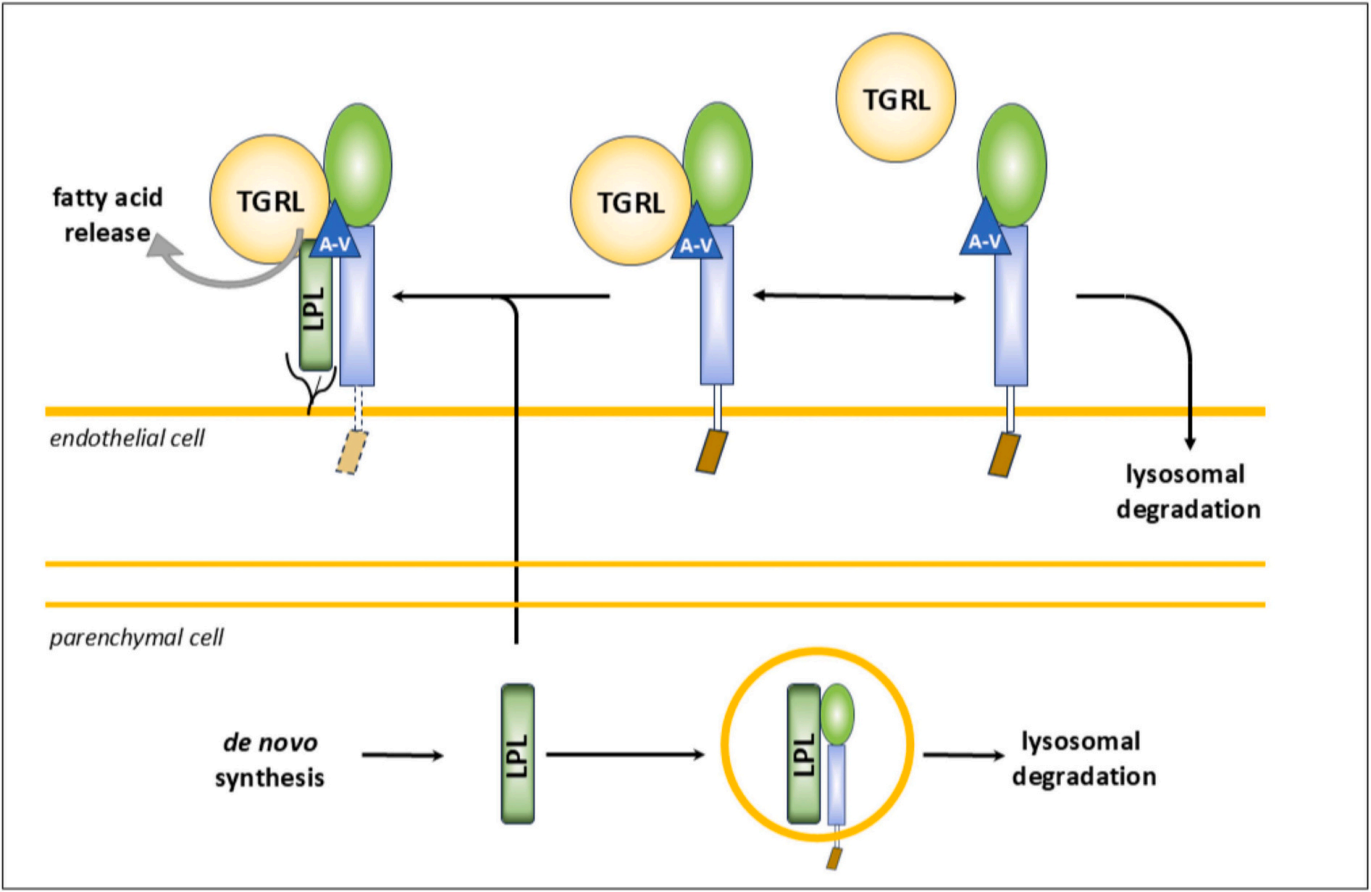
LR11 affects the metabolism of triglyceride-rich lipoproteins. Binding of LPL to LR11 in adipocytes leads to reduced expression of LPL on endothelial cells, and hence to reduced lipolysis of triglycerides in plasma triglyceride-rich lipoprotein particles. In addition, relative shortage of ApoA-V due to LR11-mediated internalization and degradation may reduce binding of TGRL to the LR11-LPL complex and LPL activation. Elevated levels of circulating TGRL may further increase LR11 expression in liver and endothelial cells [39].

Vongpromek et al. [39] provided evidence indicating involvement of LR11 in TGRL metabolism. They showed that *ldlr*-knockout mice displayed increased plasma sLR11 levels when fed a high-fat diet. LR11 may bind TGRLs through interacting with ApoE [20] or ApoA-V [23], [92]. Binding and uptake of ApoE-containing lipoproteins, such as β-VLDL, was mediated by LR11 in LR11-overexpressing Chinese hamster ovary cells [20]. Nilsson and colleagues [23], [92] identified ApoA-V as an additional ligand for LR11. ApoA-V has been proposed to activate membrane-bound LPL [93], thereby increasing lipolysis of triglycerides in VLDL and chylomicrons and consequently decreasing plasma TGRL levels. Nilsson et al. [92] have shown that ApoA-V binding to LR11 on LR11-expressing HEK293 cells results in rapid internalization of the complex, followed by transportation to the TGN. Subsequently, only a small amount of ApoA-V is recycled to the cell surface. Plasma ApoA-V levels range between 150 and 200 ng/ml, which corresponds to only approximately one ApoA-V molecule per 20 VLDL-particles. In this way, LR11 further reduces the availability of ApoA-V at the cell surface leading to reduced LPL activity and chylomicron binding. Merkel et al. [93] suggested ApoA-V activates LPL by guiding TGRLs to membrane-bound LPL. Thus, the bridging of ApoA-V in LR11/TGRL-binding may also include a complex with LPL [26]. Support for this hypothesis is provided by Klinger et al. [22], who revealed that LR11 can bind LPL directly. Analysis of HEK293 cells transfected with LPL and LR11 showed that the complex of LR11 and LPL is mainly located within vesicles. Co-localization of LPL with the lysosomal marker LAMP1 suggests these vesicles are directed to lysosomes. LPL was reduced in LR11 expressing cells compared to LR11 deficient cells as well as in cells that contained a non-binding LR11 variant [22]. Therefore, it is hypothesized that newly synthesized LPL binds LR11 already within the TGN. This leads to transport into lysosomes and direct degradation of LPL and consequently to reduced levels of LPL on the plasma membrane (Fig. 3). Because the interaction of LR11 with ApoA-V and LPL is investigated separately, it might be interesting to evaluate the effect of LR11 expression on complex formation with both ApoA-V and LPL to fully elucidate this role of LR11 on lipoprotein metabolism. Moreover, research on the roles of both ApoA-V and LPL in LR11-mediated lipoprotein regulation in cell types that could be associated with (related) CVD may be valuable.

On the other hand, TGRLs were found to elevate sLR11 and LR11 levels in a human hepatoma cell line and human aortic SMCs and ECs in vitro [39], [94]. In vivo, sLR11 derived from these cells may further induce SMC migration and proliferation. Elevated levels of LR11 following increased levels of circulating TGRLs may also result in reduced levels of ApoA-V and LPL. This could lead to higher levels of TGRL and subsequently further elevated LR11. Eventually, this vicious circle of increasing levels of TGRL and LR11 may increase the risk of CVD.

## 6 LR11 and obesity, a risk factor for CVD

Several studies have indicated a role of LR11 in body adiposity. Genomic wide association studies revealed a link of the *sorl1* and *SORL1* gene with obesity, body mass index (BMI), visceral adipose/subcutaneous adipose-ratio and waist circumference in mice and humans, respectively [95], [96], [97]. Weight loss in both overweight and obese patients with diabetes and patients who underwent gastric bypass resulted in reduced plasma levels of sLR11 [54], [98], which paralleled lowering of circulating triglycerides [39]. Overweight and obesity (BMI above 25 and 30 kg/m^2^, respectively) are classical risk factors for CVD [99]. An increased visceral and perivascular fat mass results in a chronic state of low grade inflammation, which negatively affects endothelial and smooth muscle cell function and hence, increases atherogenesis [100], [101]. Thus, a possible role of LR11 in the pathogenesis of obesity may contribute to the risk of the development and progression of CVD.

Both Whittle et al. [98] and Schmidt et al. [102] demonstrated that mice lacking LR11 do not develop diet-induced obesity. On the other hand, LR11 overexpressing mice that were fed a high-fat diet showed less triglyceride hydrolysis, hypertrophy of white adipose tissue (WAT) and enhanced lipid accumulation in muscle and liver, compared to wild-type mice and knock-out mice [102]. LR11 was shown to facilitate diet-induced expansion of the adipocyte precursor cell pool in visceral white adipose tissue of juvenile mice, which may lead to increased adiposity in adulthood [103]. The LR11-knockout mice on a high fat diet had an increased metabolic rate compared to wild-type mice, which was accompanied by improved fasting glucose, insulin and triglyceride levels. Furthermore, the LR11 deficient mice show a conversion of white to brown adipose tissue (BAT). Both groups did not find differences in LPL level or LPL activity, which indicates that LR11 affects other pathways in lipid metabolism [97], [102]. During differentiation in vitro of preadipocytes isolated from human and mouse subcutaneous fat to beige and brown adipocytes, the expression of LR11 mRNA and sLR11 protein was drastically suppressed, suggesting that sLR11 plays a role in the white-to-brown adipocyte trans-differentiation [104].

The absence of obesity in LR11 knock-out mice may be explained by the increased BAT activity and thus increased thermogenesis, which plays a crucial role in the consumption of lipids to produce heat. That increased thermogenesis is involved in the development of obesity is supported by the fact that detectable BAT mass and activity are negatively associated with age, BMI and diabetes [105]. The browning of WAT in LR11-deficient mice is accompanied by the increased expression of uncoupling protein 1 (Ucp1) and of bone morphogenetic protein 8b (Bmp8b) [98]. Furthermore, in vitro assays using differentiated adipocytes from wild-type or LR11 knock-out mice, showed that adipocytes from LR11 knock-out mice have greater thermogenic capacity, which was reduced by incubation with sLR11 [98]. Moreover, sLR11 treatment of wild-type adipocytes decreased intracellular lipolysis. sLR11 inhibits thermogenesis via inhibition of the BMP/Smad signaling pathway [98]. This pathway plays a key role in the regulation of BAT activity and browning of WAT. Whittle et al. [98] showed that sLR11 can bind to the BMP receptor, which results in reduced thermogenic signaling. However, whether this binding occurs already within the TGN or at the cell surface, and how this is regulated, remain to be clarified.

Schmidt et al. [102] also revealed an effect of LR11 on intracellular lipolysis. They showed a reduced free fatty acid production in gonadal fat explants of LR11-overexpressing mice compared to wild-type or LR11-deficient mice, indicating a decreased lipolytic activity in LR11-overexpressing mice. Further examination of the adipocytes showed a greater relative reduction of the free fatty acid release by LR11-overexpressing adipocytes when treated with insulin, compared to adipocytes from wild-type or LR11 knock-out mice. These results indicate an enhanced response to insulin of adipocytes from LR11-overexpressing mice, and thus enhanced levels of the activated insulin receptor. There was a positive correlation between number of insulin receptors and levels of LR11, with the highest levels in LR11-overexpressing mice [102]. The insulin receptor induces phosphodiesterase 3β activity, resulting in a conversion of cAMP to AMP. cAMP is required for activation of protein kinase A, which in adipocytes induces the degradation of triglycerides to glycerol. By lowering cAMP, intracellular lipolysis is inhibited. Increased levels of the insulin receptor at the cell surface may be explained by altered sorting of the insulin receptor by LR11. Experiments with LR11-expressing CHO cells indicated that internalized insulin receptor molecules are recycled to the cell surface, escaping from degradation in the lysosomal compartments [102].

Concluding, Whittle et al. [98] proposed regulating features of LR11 on thermogenesis in WAT. In case of LR11 deficiency, this induces brown adipocyte recruitment, hypermetabolism and less lipid accumulation that will phenotypically present in leanness. In the presence of LR11 or sLR11, expression of the insulin receptor on the cell membrane is increased, which leads to stronger insulin-mediated suppression of intracellular lipolysis [102]. Both groups indeed showed a positive association between LR11 and body weight. On the other hand, LR11 or sLR11 reduces LPL activity thereby increasing levels of circulating TGRLs, which in turn could induce LR11 expression. By increasing TGRLs, LR11 indirectly contributes to the risk of CVD.

## 7 LR11 as a therapeutic target

That sLR11 is involved in the development of cardiovascular diseases in vivo is primarily based on clinical studies on a wide variety of diseases with different pathophysiology (Table 1). This notion is supported by the fact that increases in sLR11 levels correlate with the severity of disease, and in some studies with other well-established disease markers, such as hsCRP, TNFα, AGEs or HbA1c, as well (Table 2). Nevertheless, the level of evidence for such a role obtained from these human studies, being mainly observational in nature and including only small number of patients, remains rather limited. Much stronger evidence comes from the many experimental studies on laboratory animals and cultured cells, which gave insight into the mechanisms by which sLR11 may affect vascular remodeling, either directly via SMC migration, or indirectly via interaction with fat and lipoprotein metabolism. LR11 may therefore be an interesting therapeutic target to reduce the risk of vascular diseases, in general, or the residual risk of CVD that remains despite patients receiving statin treatment [106] However, it should be taken into consideration that low LR11 expression in the brain is associated with early and late onset Alzheimer's disease [14], [15]. Thus, therapeutics targeting LR11 should not be able to cross the blood-brain barrier and affect LR11 levels within the brain.

Treatment of cuff-injured mice with an LR11 antibody resulted in a reduced intima-media thickness and fewer intimal SMCs [73]. Antibodies will primarily target circulating sLR11 and surface-expressed LR11 in the vascular bed, and are unlikely to reach the brain. In contrast, inhibition of LR11 expression by allele-specific oligonucleotides (ASO) or small interfering RNAs (siRNA) are expected to primarily affect the sorting activity of intracellular LR11 in all expressing cells of the body, possibly also of the brain. In fact, the commonly used HMG-CoA reductase inhibitors, statins, appear to reduce vascular SMC migration and proliferation [63], [107], and thereby suppressing neointimal thickening [63]. Jiang et al. [63] have suggested that pitavastatin reduces SMC migration via the inhibition of the LR11 expression and the LR11/uPAR pathway. Whether this holds for other statins as well is unknown. TGRLs may also be involved here, since statins reduce circulating levels of TGRL via lowering of VLDL secretion. Not all patients receive optimal statin treatment to achieve LDL-cholesterol target levels, and perhaps even higher statin doses are required to optimally reduce LR11 levels. However, lipophilic and hydrophilic statins can cross the blood-brain barrier by passive diffusion or via active transport, respectively, with greater permeability for the lipophilic statins. Therefore, statins may affect LR11 levels within the brain. Nevertheless, long term use of lipophilic as well as hydrophilic statins appears to reduce the risk of Alzheimer's disease, which is likely attributable to their lipid lowering, anti-oxidant and anti-inflammatory functions [108], [109].

Targeting LR11 in monocytes and macrophages may be another way to reduce the risk of CVD. McCarthy et al. [109] showed that conjugated linoleic acids (CLA) reduce LR11 levels in monocytes in vitro and in infiltrated macrophages in vivo, which might play a role in the CLA-mediated regression of atherosclerosis. CLA are ligands for the athero-protective peroxisome proliferator activator receptors (PPARs). The effect of CLA on LR11 levels is, at least partly, mediated by PPAR-γ activation [110]. Lowering levels of LR11 in monocytes may lead to reduced monocyte migration and infiltration into the vessel wall and hence to reduced atherogenesis. Furthermore, Lee et al. [111] proposed a neuroprotective role of CLA in neurons by inhibiting the accumulation of beta-amyloid in a neuronal cells.

Conspicuously, sorting by LR11 either results in more cell surface expression or to less expression of membrane proteins, as exemplified here by the uPAR, PDGFR and insulin receptor, and LPL and ApoA-V, respectively, as a consequence of decreased internalization, increased trafficking towards lysosomal degradation, or increased recycling towards the plasma membrane. These roles of LR11 in protein trafficking may be cell-type specific, and depend on the pattern of adaptor molecules that interact with the cytoplasmic tail of LR11. Alternatively, it may depend on the presence or absence of the cytoplasmic tail, whether it involves the intact LR11 protein or the soluble sLR11. ADAM17-induced shedding of the ectodomain occurs on the cell surface, which may prevent sLR11 from being internalized on its own. In addition, circulating sLR11 has similar effects as endogenous LR11 on vascular SMC proliferation and migration, and potentially acts throughout the entire vascular bed. It is therefore worthwhile to study the effect of ADAM17 inhibition. ADAM17 is expressed on endothelial and myeloid cells [112]. Inhibition of endothelial ADAM17 expression can suppress atherogenesis, whereas inhibition in myeloid cells can lead to increased plaque stability. Several small molecule inhibitors and monoclonal antibodies have successfully been tested in cell cultures, but none have succeeded yet in clinical trials [112].

It should be stressed, however, that most available evidence for a role for sLR11 in vascular remodeling leading to atherosclerosis and other cardiovascular diseases is derived from experimental and preclinical studies. Clinical translation therefore remains to be established.

## 8 Conclusion

The number of studies presenting associations of sLR11 with CVD is rising, showing that sLR11 might be an interesting biomarker for these types of diseases. However, the evidence supporting sLR11 as a biomarker remains limited and is largely observational. Prospective validation studies and assay standardization are needed before it can be implemented in routine clinical practice. (s)LR11 may play an active role in inducing SMC migration and proliferation in atherosclerosis, which is the cause of most CVDs. Furthermore, the receptor may be involved in TGRL-metabolism, but the mechanism requires further examination. Much of the evidence is derived from research on cells in culture, thus whether (s)LR11 indeed has a causal role in atherosclerosis and cardiovascular diseases still remains to be established. Statins and conjugated linoleic acids reduce the progression of atherosclerosis and reduce the expression of (s)LR11, suggestive of a key role in the pathology of this disease. However, statins are athero-protective also by their lipid lowering, anti-inflammatory, endothelial-protective and plaque-stabilizing effects. Elevated sLR11 levels are associated with obesity and diabetes indicating a role of LR11 in these diseases as well. In both diseases, levels of LR11 may be induced by increased levels of circulating TGRLs, though TGRLs may also enhance LR11 expression.

**Fig. 4:**
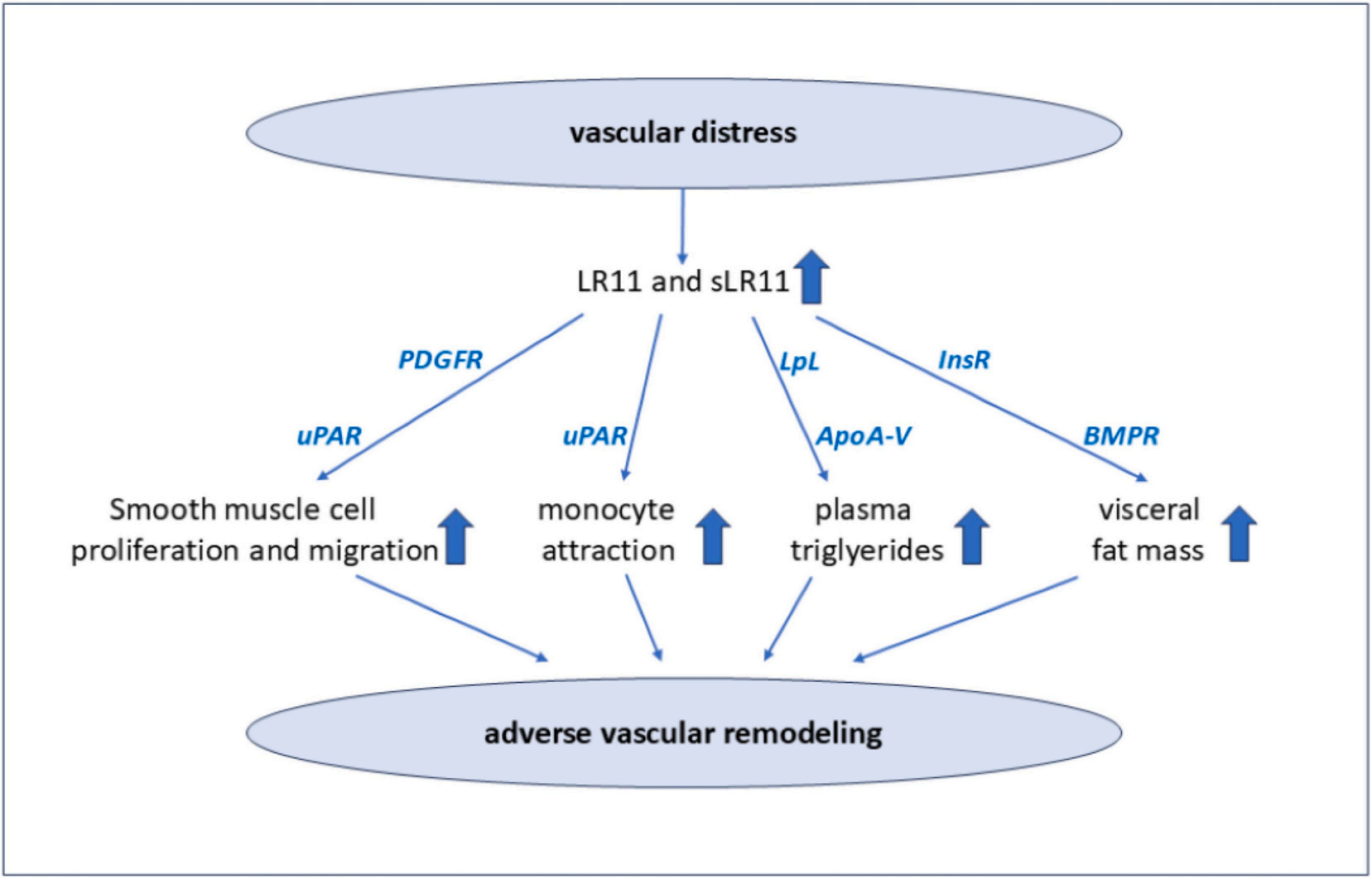
Vascular distress leads via upregulation of LR11 to adverse vascular remodeling. Receptors involved are indicated.

The picture that emerges in this review is that any form of vascular distress, be it inflammation, hypertension or cardiometabolic disturbances, leads to increased expression of LR11 and sLR11 in the vascular bed (Fig. 4). Elevated LR11 and sLR11 induce SMC proliferation and migration into the intima, disturb lipolysis of TGRLs and increase fat accumulation in visceral and perivascular adipose tissue, which further increase vascular remodeling through inflammation, monocyte attraction and lipid accumulation. This vicious cycle eventually leads to atherosclerosis.

Further research is required to elucidate the roles of LR11 and sLR11 in these CVD risk-enhancing diseases. Most of the functional studies on SMC migration and lipolysis have been conducted in vitro using cell cultures and animal studies, thus the in vivo role of LR11 in the development of CVDs remains to be confirmed. Pitavastatin has been shown to reduce the expression of (s)LR11 [63]. It should be tested whether this holds for the other statins as well. Is LR11 expression in brain, or in specific brain regions, affected by statin treatment? Is the effect of statins on (s)LR11 expression also seen with other cholesterol lowering medications, such as PCSK9 inhibitors, or is (s)LR11 lowering one of the many pleiotropic effects of statins? Is the dose-response curve of statins for lowering of (s)LR11 similar to that for plasma cholesterol, or are higher statin doses required to optimally affect (s)LR11 levels? Is the relationship between (s)LR11 and vascular remodeling, atherosclerosis and cardiometabolic disorders affected by statin treatment? Furthermore, which of the effects of increased LR11 expression on SMC migration, TGRL metabolism, monocyte attraction and visceral fat mass are primarily induced by the soluble form and can be prevented by inhibitors of ADAM17? Can adverse vascular remodeling be slowed or prevented by inhibition of LR11 expression by intensified statin therapy, conjugated linoleic acid supplementation, by monoclonal antibodies against LR11, or through prevention of LR11 shedding by ADAM17 inhibition?

## CRediT authorship contribution statement

**Meander Kloosterman:** Writing – original draft. **Ranitha Vongpromek:** Writing – review & editing. **Edith C.H. Friesema:** Writing – review & editing, Validation. **Wichor Bramer:** Writing – review & editing, Software, Resources, Formal analysis. **Adrie J.M. Verhoeven:** Writing – review & editing, Visualization. **Monique T. Mulder:** Writing – review & editing, Supervision, Project administration, Conceptualization.

## Declaration of competing interest

The authors declare that they have no known competing financial interests or personal relationships that could have appeared to influence the work reported in this paper.
